# Neuromorphic Sentiment Analysis Using Spiking Neural Networks

**DOI:** 10.3390/s23187701

**Published:** 2023-09-06

**Authors:** Raghavendra K. Chunduri, Darshika G. Perera

**Affiliations:** Department of Electrical and Computer Engineering, University of Colorado Colorado Springs, 1420 Austin Bluffs Parkway, Colorado Springs, CO 80918, USA; raghavkumar1988@gmail.com

**Keywords:** neuromorphic computing, artificial neural network, natural language processing, sentiment analysis, spiking neural networks, SpiNNaker

## Abstract

Over the past decade, the artificial neural networks domain has seen a considerable embracement of deep neural networks among many applications. However, deep neural networks are typically computationally complex and consume high power, hindering their applicability for resource-constrained applications, such as self-driving vehicles, drones, and robotics. Spiking neural networks, often employed to bridge the gap between machine learning and neuroscience fields, are considered a promising solution for resource-constrained applications. Since deploying spiking neural networks on traditional von-Newman architectures requires significant processing time and high power, typically, neuromorphic hardware is created to execute spiking neural networks. The objective of neuromorphic devices is to mimic the distinctive functionalities of the human brain in terms of energy efficiency, computational power, and robust learning. Furthermore, natural language processing, a machine learning technique, has been widely utilized to aid machines in comprehending human language. However, natural language processing techniques cannot also be deployed efficiently on traditional computing platforms. In this research work, we strive to enhance the natural language processing traits/abilities by harnessing and integrating the SNNs traits, as well as deploying the integrated solution on neuromorphic hardware, efficiently and effectively. To facilitate this endeavor, we propose a novel, unique, and efficient sentiment analysis model created using a large-scale SNN model on SpiNNaker neuromorphic hardware that responds to user inputs. SpiNNaker neuromorphic hardware typically can simulate large spiking neural networks in real time and consumes low power. We initially create an artificial neural networks model, and then train the model using an Internet Movie Database (IMDB) dataset. Next, the pre-trained artificial neural networks model is converted into our proposed spiking neural networks model, called a spiking sentiment analysis (SSA) model. Our SSA model using SpiNNaker, called SSA-SpiNNaker, is created in such a way to respond to user inputs with a positive or negative response. Our proposed SSA-SpiNNaker model achieves 100% accuracy and only consumes 3970 Joules of energy, while processing around 10,000 words and predicting a positive/negative review. Our experimental results and analysis demonstrate that by leveraging the parallel and distributed capabilities of SpiNNaker, our proposed SSA-SpiNNaker model achieves better performance compared to artificial neural networks models. Our investigation into existing works revealed that no similar models exist in the published literature, demonstrating the uniqueness of our proposed model. Our proposed work would offer a synergy between SNNs and NLP within the neuromorphic computing domain, in order to address many challenges in this domain, including computational complexity and power consumption. Our proposed model would not only enhance the capabilities of sentiment analysis but also contribute to the advancement of brain-inspired computing. Our proposed model could be utilized in other resource-constrained and low-power applications, such as robotics, autonomous, and smart systems.

## 1. Introduction

In recent years, the Artificial Neural Networks (ANNs) domain has witnessed a significant adaptation of Deep Neural Networks (DNNs) across several fields, such as machine learning, computer vision, artificial intelligence, and natural language processing (NLP). DNNs are capable of accurately performing a wide range of tasks by training on massive datasets [1]. However, the energy consumption and computational cost required for training large volumes of datasets and for deploying the resulting applications have been of less importance; thus, they have been overlooked [2,3]. The DNNs typically consume high power and require large data storage [4,5]. Although there have been significant advancements in ANNs, ANNs were unable to achieve the same level of energy efficiency and online learning ability as biological neural networks [6]. Drawing inspiration from brain-inspired computing, one potential solution to address the issue of high-power consumption is to use the neuromorphic hardware with Spiking Neural Networks (SNNs). SNNs, often considered the third generation of neural networks, are emerging to bridge the gap between fields such as machine learning and neuroscience [7].

Unlike traditional neural networks that rely on continuous-valued signals, the SNNs work in continuous time [8]. In SNNs, the neurons communicate with each other using discrete electrical signals called spikes. Spikes model the behavior of the neurons more accurately and more biologically plausible than ANNs, thus making SNNs more energy efficient and computationally powerful than ANNs [9]. The neuron models of ANNs and SNNs differ from each other. For instance, ANNs do not have any memory and use sigmoid, tanh, or rectified linear unit (ReLU) as computational units, whereas SNNs have memory and use non-differentiable neuron models. Typically, large-scale SNN models consume high power and require high execution time when utilized/executed on classical Von Neumann architectures [10]. Hence, there is a need for high-speed and low-power hardware for executing large-scale SNN models. In this regard, existing neuromorphic platforms, such as SpiNNaker [11], Loihi [12], NeuroGrid [13], and TrueNorth by IBM [14], are expected to advance the applicability of large-scale SNNs in several emerging fields by offering energy-efficient high-speed computational solutions. SNNs have the functional similarities to biological neural networks, allowing them to embrace the sparsity and temporal coding found in biological systems [15]. However, SNNs are difficult to train because of their non-differentiable neuron models. In terms of speed performance, SNNs are inferior to DNNs. Nevertheless, due to the low power traits, SNNs are considered more efficient than DNNs [6].

Considering the aforementioned, in this research work, we propose a novel and unique neuromorphic NLP sentiment analysis model based on the SNNs, deployed on the SpiNNaker neuromorphic platform. Our proposed sentiment analysis model is created in such a way to be energy efficient and also to be faster than the ANN-based NLP models, while predicting the sentiment. Our experimental results illustrate that our proposed model is energy efficient and consumes low power when executed on the Internet Movie Database (IMDB) dataset to predict the reviews based on user inputs. Our results and analysis also demonstrate that our proposed model is more efficient and effective compared to the existing ANN-based sentiment analysis models in the published literature. In this paper, we make the following contributions:

- We introduce a novel, unique, and efficient sentiment analysis model for neuromorphic hardware using SNNs. Our proposed model is highly accurate, while detecting text and predicting the sentiment.
- Our proposed model is adaptable, energy efficient, and computationally effective. These traits make our model well suited for areas where energy efficiency is crucial and resources are scarce.
- We perform experiments to evaluate the feasibility and efficiency of our proposed model. Our results and analysis demonstrate that our model is efficient and accurate compared to the ANN-based sentiment analysis model.

Our paper is organized as follows. Section 2 provides an overview of SNNs and their applications in various fields. In Section 3, we discuss and present our proposed SNN model in detail, highlighting its unique features. In Section 4, we present our experimental results and analysis, including the comparison of speed performance and energy consumption of our proposed SNN model with traditional existing ANN models. In Section 5, we summarize, conclude, and discuss the potential impacts of SNNs in various fields and our future directions.

## 2. Background

In this section, we provide brief descriptions of the SNNs, ANNs to SNNs conversion, SpiNNaker computing models, and NLP sentiment analysis.

### 2.1. Spiking Neural Networks (SNNs)

As stated in [16], the SNNs are considered the third generation of neural networks, which communicate through a sequence of discrete electrical events called “spikes” that takes place at a point of time. The SNN models are generally expressed in the form of differential equations [17]. The structure of spiking neurons in the SNN model is similar to the structure of the ANN neuron; however, their behavior is different. SNNs are widely used in various applications, including brain–machine interface, event detection, forecasting, and decision making [18,19]. The difference between the SNNs and the ANNs, in terms of various parameters, are presented in Table 1, sourced from [9].

**Figure 1:**
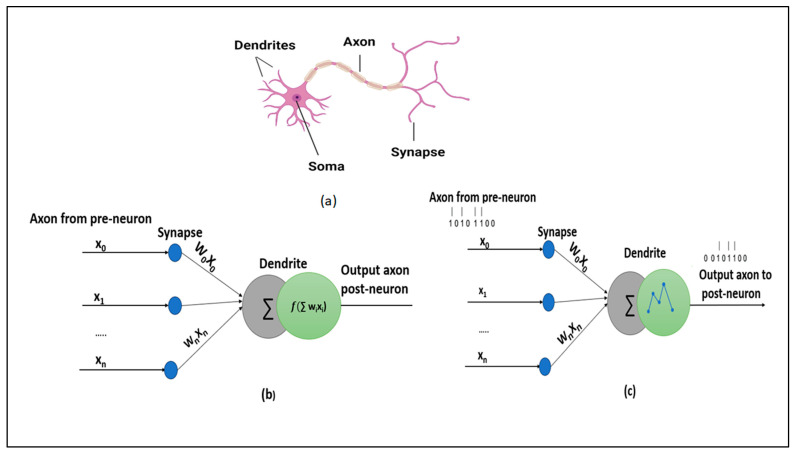
Schematic representations of (**a**) biological neural network, (**b**) artificial neural network, and (**c**) spiking neural network.

The spiking neuron models are distinguished based on the biological plausibility and computational capabilities [20,21,22]. Typically, spiking neuron models are selected based on specific user requirements. In this subsection, we briefly discuss and present the most commonly used spiking neuron models, including Hodgkin–Huxley (HH), Integrate-and-Fire (IF), Spike Response model, and Izhikevich model, which are prominent models that are widely used in various applications. These models differ from each other in terms of biological characteristics and computational complexity. Figure 1 illustrates the schematics of a biological neural network, ANN and SNN [17].

#### 2.1.1. Hodgkin–Huxley Model

The Hodgkin-Huxley (HH) model is the first biological model of spiking neuron [23]. This model explains how the neuron actions are initiated and propagated. In this model, the electric current through the membrane potential in terms of mathematical description can be computed using Equation (1) [23]:

(1) $$I=C\frac{dv}{dt}+G_{Na}m^{3}h\left(V-V_{Na}\right)+G_{k}n^{4}\left(V-V_{k}\right)+G_{L}\left(V-V_{L}\right)$$

where *I* represents the external current, and *C* represents the capacitance of the circuit.

The parameter modelling conductance of sodium, potassium, and leakage channels are represented as *G_Na_*, *G_k_* and *G_L_*, respectively, whereas *V_Na_*, *V_k_* and *V_L_* are called the reverse potentials. The gating parameter *n* is used to control the potassium channel, whereas *m* and *h* control the sodium channel. For this model, Equations (2)–(4) are used to determine *m, n,* and *h* parameters.

(2) $$\frac{dm}{dt}=\alpha_{m}\left(V\right)\left(1-m\right)-\beta_{m}\left(v\right)m$$

(3) $$\frac{dn}{dt}=\alpha_{n}\left(V\right)\left(1-n\right)-\beta_{m}\left(v\right)n$$

(4) $$\frac{dh}{dt}=\alpha_{h}\left(V\right)\left(1-h\right)-\beta_{h}\left(v\right)h$$

The HH model is computationally expensive due to the differential equations of *m*, *n*, and *h* parameters. Also, the HH model requires nearly 1200 floating point computations [23]; hence, it is not suitable for applications that require large-scale neural network simulation/execution. A detailed description of the HH model and the corresponding equations can be found in [23].

#### 2.1.2. Izhikevich Model

Another neuromorphic model was proposed by Izhikevich [24]. It is a two-dimensional biologically plausible spiking neuron model which exhibits the complete behavior of the neurons. The Izhikevich model describes the hippocampal neurons, which are suitable for large-scale simulations [24]. This model is represented using two differential Equations (5) and (6), below, whereas Equation (7) is used to adjust the membrane voltage (*v*) and recovery variable (*u*).

(5) $$\frac{dv\left(t\right)}{dt}=0.04v^{2}+5v+140-u+I\left(t\right)$$

(6) $$\frac{du\left(t\right)}{dt}=a\left(bv-u\right)$$

(7) $$u\left(vv_{th}\right)=c\approx and\approx u\left(v>v_{th}\right)=u+d$$

In the above equations, the membrane potentials of the neurons are represented using *v*, whereas *u* represents the membrane recover variable, which accounts for the iconic current activation of K+ and inactivation of Na+. In the Izhikevich model [24], by changing the injected stimulus and tuning the parameters, multiple spiking patterns can be produced. A detailed description of the Izhikevich model and the corresponding equations can be found in [24].

#### 2.1.3. Integrate-and-Fire Model

The Integrate-and-Fire (IF) model is the simplest SNN model, which works by integrating the input spikes to the membrane portal with a predefined threshold [25]. In this case, if the threshold is reached, an output spike is generated. The generation of the output spike changes the membrane portal to a resting state. This model is determined by Equation (8), as follows [25]:

(8) $$C_{m}\frac{dv}{dt}=I\left(t\right),v\leftarrow v_{rest}\approx when\approx v\geq v_{th}$$

where *C_m_* is the membrane capacitance, *v_th_* is the threshold, *v* is the membrane potential, and *v_rest_* is the resting potential.

The IF model consumes less power when compared to other SNN models [25]. The Leaky Integrate-and-Fire (LIF) model is an important type of integrate–fire (IF) model with leaks added to the membrane potential. The LIF model is defined using Equation (9) [25].

(9) $$T_{leak}\frac{dv}{dt}=\left[V\left(t\right)-v_{rest}\right]+r_{m}I\left(t\right),v\leftarrow v_{rest}\approx when\approx v\geq v_{th}$$

where *T_leak_* = *r_m_c_m_* is the membrane time constant, and *r_m_* is the membrane’s resistance.

Due to the IF model’s accuracy in terms of replicating spiking behavior, as well as the simulating speed and low computational cost, the IF model is widely used in applications that require large-scale neural network simulations. A detailed description of the IF model and the corresponding equations can be found in [25].

In this research work, for our proposed model we use the IF spiking neurons, mainly because of their low computational cost and low power consumption. The IF model is one of the most popular models utilized to understand the relationship between the variability of inputs to the neurons, and the variability of their outputs [26]. In addition, the IF model is used to understand the properties of large neural networks. More complex types of IF models, such as quadratic integrate and fire, adaptive exponential integrate and fire, and exponential integrate and fire, can be found in the literature [27,28,29].

#### 2.1.4. Spike Response Model (SRM)

The Spike Response Model (SRM) is another bio-inspired spiking neuron model, which describes the effect of input spikes on the membrane portal more precisely [30]. Similar to the IF model, the SRM model also generates the output spikes, whenever the threshold of the internal membrane portal is reached. Unlike the LIF model, the SRM model uses response kernels for voltage potential. The mathematical formula of the SRM model is expressed in Equation (10) [31].

(10) $$v\left(t\right)=\eta \left(t-\hat{t}\right)+\int_{-\infty }^{+\infty }k\left(t-\hat{t},s\right)I\left(t-s\right)ds$$

where *v*(*t*) is the neurons internal potential, $\hat{t}$ is the emission time of the last neuron output spike, η describes the state of action potential, *k* is the linear response to an input spike, and *I*(*t*) represents the external or stimulating current. A detailed description of the SRM model and the corresponding equations can be found in [30].

The SRM model offers low computational cost and requires 50 floating-point computations per 1 ms simulation [21]. Compared to other spiking neuron models, the SRM model provides poor biological plausibility, and it is computationally complex when utilized in digital system applications [21]. In Table 2, we present the comparison of various SNN models, with respect to floating-point operations per second (FLOPS) and computational complexity [21].

### 2.2. ANN to SNN Conversion

The ANNs are used extensively for solving several tasks in various fields, such as machine learning and artificial intelligence. In this case, deep learning develops large neural networks with millions of neurons that span up to thousands of layers. These large neural networks have proven to be effective while solving several complex tasks, including video classification, object detection and recognition, etc.; however, these networks require massive computational resources [32,33,34,35]. The development of SNNs is mainly to address the challenge associated with massive computational resources. The SNNs perform similar tasks with less computational resources and with low energy consumption. In SNNs, all the computations are event-driven, and operations are sparse. In this case, the computations and operations are performed only when there is a significant change in the input. Typically, training a large SNN is a difficult task; thus, an alternative approach is to take a pre-trained ANN network and convert it into SNNs [1]. Existing ANN-to-SNN conversion methods in the literature primarily focus on converting ReLu to IF neurons. In the proposed model, we utilize an ANN-to-SNN conversion method proposed by [36], which uses the weights of the ANN that replaces the analog (rate-based) neurons with integrate-and-fire spiking neurons. For our proposed model, during the simulation, the average firing rate of SNN neurons will gradually approximate the activations of corresponding original neurons. Additionally, for our model, the deep SNNs have produced comparable results with ANNs after the conversion and offer promising solutions to the energy-efficiency problems in the ANNs during the time of deployment. 

**Figure 2:**
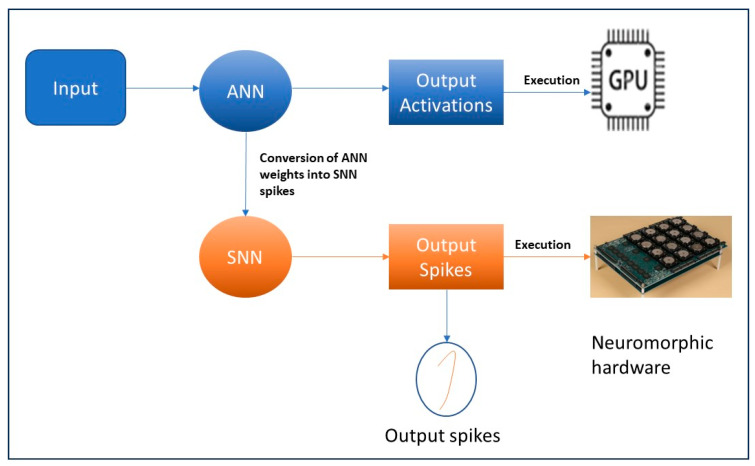
Overview of our ANN-to-SNN conversion.

An overview of our ANN-to-SNN conversion is illustrated in Figure 2. The process of converting from ANN to SNN involves transferring the trained ANN settings that use ReLU activations to an SNN with an identical structure, as depicted in Figure 2. This approach enables the SNN to achieve exceptional performance while requiring minimal computational resources. Initially, the ANN model is trained with the given inputs, and the weights are saved. Typically, traditional trained ANN models are being executed on GPUs, as illustrated in top modules in blue (in Figure 2). For our proposed model, the trained ANN model and the weights are converted into SNN spikes and executed on neuromorphic hardware, as demonstrated in the bottom modules in orange (in Figure 2). 

### 2.3. Neuromorphic Hardware

The neuromorphic hardware for SNNs is categorized into analog, digital, or mixed-signal (analog/digital) designs [37]. Many neuromorphic hardware platforms with varying configurations have emerged to manage large-scale neural networks. From these neuromorphic platforms, fully digital and mixed-signal hardware, such as IBM TrueNorth, NeuroGrid, BrainScaleS, Lohi, and SpiNNaker, are some of the commonly used platforms among several applications [38]. A detailed description of the neuromorphic hardware platforms can be found in [38]. 

In Table 3, we present various features/characteristics of existing neuromorphic hardware platforms in the published literature. These details/comparisons provide insight into different SNN architectures and learning mechanisms in the neuromorphic computing domain. This information is relevant and important for us to select and utilize a suitable neuromorphic platform for our current as well as future research work. In our proposed work, we use the SpiNNaker neuromorphic platform. Hence, a detailed description of the SpiNNaker system is discussed in the following subsection. A more detailed description of these neuromorphic platforms (as in Table 3) can be found in [9].

### 2.4. SpiNNaker

The SpiNNaker was designed by the Advanced Processor Technologies Research Group (APT), from the Department of Computer Science at the University of Manchester [39]. It is composed of 57,600 processing nodes, each with 18 ARM9 processors (specifically ARM968), 128 MB of mobile DDR-SDRAMs, totaling 1,036,800 cores, and over 7 TB of RAM [40,41]. The SpiNNaker is an SNN architecture designed to simulate large-scale SNNs. The main component of the SpiNNaker system is the SpiNNaker chip, whose main focus is to provide the required scalability and flexibility to perform experiments with neuron models. Based on brain-inspired computing, the objective of the SpiNNaker system is to design the neural architecture model of the human brain which is made up of approximately 100 billion neurons connected by trillions of synapses [39]. The SpiNNaker machine is a collection of low-power processors, which can simulate/execute a small number of neurons and synapses in real time. In this case, all the processors are interconnected by a high-speed network [42]. The high-speed network allows the processors to communicate with each other, while distributing the computation load for simulating a large neural network. The main advantage of the SpiNNaker system is its ability to simulate large-scale neural networks using an asynchronous scheme of communication [40,43], which is essential for testing brain functions and developing new neural network applications in areas such as robotics, machine learning, and artificial intelligence. The SpiNNaker system is indeed an exciting creation in the field of neural networks, and it has the potential to greatly advance the understanding of the brain and the information processing of the brain [44,45].

#### 2.4.1. Architecture of SpiNNaker Chip

As stated in [45], the SpiNNaker chip has 18 cores coupled with an external RAM controller and a Network-on-Chip (NoC). Each core comprises an ARM968 processor, a direct memory access controller, a controller for communications, a network interface controller, and other peripherals, including a timer [45]. Every core in the SpiNNaker chip runs given applications by simulating/executing a group of neurons at 200 MHz. Each core also comprises 96 kB of tightly coupled memory (TCM). In order to avoid any contention issues, this TCM is split into two: 64 kB for data (DTCM), and 32 kB for instructions (ITCM). The DTCM consists of application data, including zero-initialized data, heap, stack, and read/write. Each chip in the SpiNNaker system has 128 MB of shared memory (i.e., SDRAM), which is directly accessible by all cores in the SpiNNaker chip [45]. In this case, the memory access time varies significantly when accessing the different memories mentioned above. Hence, the following should be considered when designing applications for the SpiNNaker system.

- Faster access to DTCM at ≈5 ns/word. DTCM is only limited to the local core.
- Access to SDRAM via a bridge. Accessing SDRAM could lead to a contention issue, since more than one core in the SpiNNaker chip could attempt to access. This is a slow process with >100 ns/word.
- As a result, each core encompasses a direct memory access (DMA) controller, which is used to enable bulk transfer of data from the SDRAM core to DTCM efficiently. Although the DMCA setup introduces a fixed overhead, the data are still transferred from the processor independently at ≈10 ns/word.

The SpiNNaker is a large-scale parallel network, comprising low-power and energy-efficient processors connected by a network. Each node in the network is responsible for simulating/executing a small number of neurons and synapses [44]. Each node in the network communicates with every other node to exchange information and distribute the computation load. Each node in the network consists of processors, memory, I/O interfaces and core. Every node in the SpiNNaker architecture is constructed from one or more SpiNNaker boards, which are made up of SpiNNaker chips [11]. Currently, two production versions called SpiNN-3 and SpiNN-5, each of which has 4 and 48 chips, respectively, are available. In Table 4, we present the characteristics of various memory access of the SpiNNaker chip sourced from [11].

#### 2.4.2. Components of SpiNNaker System

The architecture of the SpiNNaker system consists of the following four major components [11].

**Processing nodes**: are the individual processors used to simulate/execute the behavior of artificial neurons and synapses.

**Interconnect fabric**: is a high-speed network used to connect the processing nodes together. Interconnect fabric allows efficient communication between the nodes, as well as efficient load distribution across the network.

**Host machine**: is a separate master computer/processor used to configure and control the SpiNNaker system. The host machine constantly communicates with the processing nodes via the network interface. The host machine also provides a user interface to set up and run the simulations.

**Software stack**: consists of a variety of software components that work together to enable the simulation/execution of neural networks on the SpiNNaker system. This software stack includes the operating system running on the processing nodes, higher-level software libraries, and tools for configuring and running the simulations.

### 2.5. PyNN

In [46], the authors introduced PyNN, which is a python interface used to define the simulations after creating the SNN model. The simulations are typically executed on the SpiNNaker machine via an event-driven operating system [46]. Using a python script, PyNN allows users to specify the SNN simulations for executions. In this case, NEST, Neuron, INI, Brian, and SpiNNaker are commonly used SNN simulators. In this research work, we use the INI and SpiNNaker simulators for our simulations. Detailed information of SpiNNaker hardware can be found in [42].

### 2.6. Sentiment Analysis Using Natural Language Processing

Sentiment analysis is a natural language processing (NLP) technique that is commonly used to identify, extract, and quantify subjective information from text data [47]. Sentiment analysis is mainly used to analyze the text and determine the sentiment score. The sentiment score can range from −1 (indicating very negative sentiment) to +1 (indicating very positive sentiment), with 0 representing the neutral sentiment [48]. Using deep-learning-based approaches to perform sentiment analysis in NLP is a popular research area. Sentiment analysis is widely employed across various fields, such as marketing, finance, and customer service, to name a few [49]. Sentiment analysis can also be used to analyze financial news and social media to predict stock prices or market trends [50]. However, with the ongoing increase in data sizes, novel and efficient models (for sentiment analysis) are needed to manage and process the massive amount of data [51]. Although the existing ANN models provide the required accuracy while classifying the data, the ANN models are not efficient in terms of energy consumption and speed-performance [52]. Therefore, in this research work, we propose a novel, unique, and efficient SNN-based sentiment analysis model to address the aforementioned issues.

## 3. Our Proposed Neuromorphic NLP Sentiment Analysis Model

In this section, we discuss and present our proposed SNN-based sentiment analysis model deployed on the SpiNNaker neuromorphic hardware. Our proposed model is created in such a way to perform a detailed sentiment analysis that involves evaluating the extent of positivity/negativity associated with a phrase or a word. We name our proposed model the Spiking Sentiment Analysis model using the SpiNNaker (SSA-SpiNNaker). For our proposed model, initially, the ANNs neural network is created by adding the input, hidden, and output layers, and incorporating the dropout to avoid overfitting with a recommended rate of 50%. Then, at every layer, the dense function is utilized to connect the units of the network fully. In the hidden layers, we utilize the rectified linear units (RELU) to train the model, whereas in the output layer, we utilize the sigmoid activation. In this case, the shape of the input at the input layer is defaulted to 10,000, and the shape of the output is defaulted to 50. For our proposed model, the input is a sequence of words, whereas the output is a binary sentiment label with either 0 or 1. In this case, the value close to 0 is considered as a negative sentiment, and a value close to 1 is considered as a positive statement. After creating the ANN model and testing its accuracy, we create the SNN model based on this ANN model.

### Creation of SNN Model from ANN

Typically, training a deep SNN and learning the synaptic weights is a difficult task compared to creating a SNN model from a pre-trained ANN model. In original ANN neural networks, the activations are real values, and the analog rate of these neurons can easily be replaced using the integrate-and-fire spiking neurons in a SNN. We utilize the following steps to perform the ANN-to-SNN conversion for our proposed SSA-SpiNNaker.

1. Firstly, a Keras model is created by extracting the relevant input information. This model serves as a common abstract stage from the input.
2. Secondly, the pre-trained ANN sentiment analysis model is converted into the SNN model using the weights of the ANN, and replaces the analog neurons by simple integrate-and-fire spiking neurons.
3. Thirdly, once the SNN conversion is completed, the resulting spiking neural network is exported for simulation, to a spiking simulator or to dedicated spiking neuron chips on the neuromorphic hardware. In our case, the SSA-SpiNNaker is deployed on the SpiNNaker hardware after the conversion.
4. Finally, our SSA-SpiNNaker model is thoroughly tested and evaluated, the accuracy is compared with our pre-trained ANN model, and the energy consumption is recorded.

We designed our proposed SSA-SpiNNaker model in python. The pseudocode for our proposed SSA-SpiNNaker model is presented in Algorithm 1. As illustrated in Algorithm 1, initially, the IMDB dataset is loaded from the Keras library. This dataset is then input to our SSA-SpiNNaker model, after importing all the necessary python modules. Then, the input dataset is divided into two: training (80%) and testing (20%). For our design, the ANN model is initialized as a sequential model and activated with the rectified linear unit (RELU) activation function. Then, we create the input layer, output layer, and the hidden layer required to train the model. Next, we compile the ANN model, train it with the given input dataset, and save the model. This saved ANN model is then converted into the SNN model by initializing the SNN model parameters, including time step, duration, batch size, and the input rate. The weights of the ANN model are converted into synapses weights. The SNN model parameters are initialized to facilitate the creation of the SNN model architecture. Based on the weight updates and spike rate, the membrane potential is updated; if the membrane potential reaches the threshold, spikes will be emitted and propagated to the next layer. Once all the spikes emission is completed, the output spikes are collected and saved as the new SNN model. Finally, we evaluate the newly created SNN model with the user inputs, and compare the accuracy with our pre-trained ANN model. The performance and accuracy of our proposed SSA-SpiNNaker model is discussed in detail in Section 4.
**Algorithm 1: Pseudocode for Our SSA-SpiNNaker Model/Algorithm**      **Data:** IMDB Dataset loaded from the Keras library       **Result:**
      Positive or Negative Review based on user inputs**Step 1:**Import all required python modulesTraining dataset (train x, train y)Test Dataset (test x, test y)**Step 2:**Initialize the ANN model as a sequential modelDefine activation functionAdd input layerAdd input layerAdd output layer**Step 3:**Compile the modelTrain the modelSave the model**Step 4:**Convert ANN weights to synaptic weightsInitialize the SNN model from saved ANN modelSetup simulation environmentInitialize simulation parameters**Step 5: foreach:** *Time step in the simulation duration* **do**                                                   **Foreach:** *For each layer in the SNN model:* **do**                                                                              **Foreach:** *For each neuron in the layer:* **do**Based on incoming spikes and weights update the neuron membrane potential.If the membrane potential exceeds firing threshold, emit a spike.By activating connected synapses, propagate the spike to next later.**Step 6:**Collect output spikesEvaluate the performance of the modelTest the model with user provided reviews

**Figure 3:**
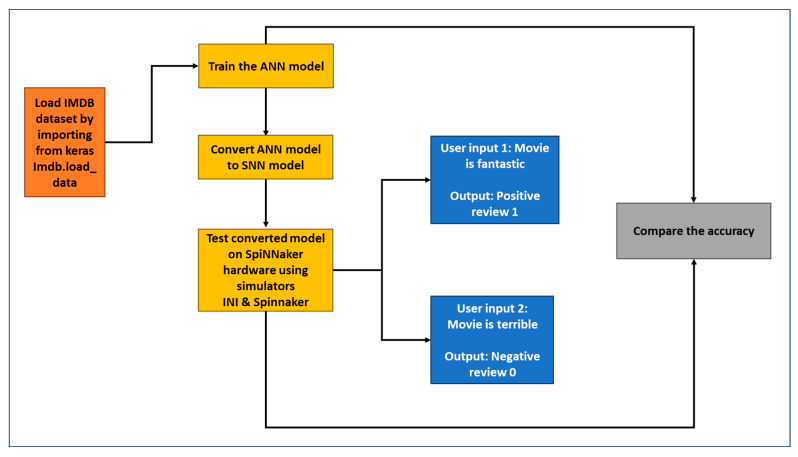
Programming model demonstrating the execution flow of our SSA-SpiNNaker model.

In Figure 3, we demonstrate the programming model of our proposed SSA-SpiNNaker model. As illustrated in Figure 3, initially, the IMDB dataset is loaded and trained using the ANN model. Then the trained ANN model is converted to the SNN model. Next, the SNN model is tested against various inputs, and the responses/results are obtained. Our proposed SSA-SpiNNaker model is compared with our pre-trained ANN model, using various simulators. For our proposed model and for the corresponding Algorithm 1, we utilize several important parameters to ensure the accuracy and efficiency of our results. The parameters used for our SSA-SpiNNaker model are as follows:

- The time step parameter represents the interval at which our proposed model processes information utilized for time resolution of spikes in milliseconds.
- The duration parameter determines the runtime of simulation of one input in milliseconds.
- The batch size parameter specifies the number of test samples that will be simulated in parallel. This parameter impacts both the computational efficiency and memory usage.
- The simulator runs the converted spiking network.
- The input-rate parameter dictates the frequency at which new data are fed into the model, mirroring the pace of data arrival in real-world applications.
- The threshold in millivolts defines the voltage at which a spike is fired.

By meticulously configuring and tuning these parameters, we strive to balance the tradeoff between precision and computational efficiency. This, in turn, will enable us to extract meaningful insights and to derive appropriate conclusions from our experiments, as presented in Section 4.

## 4. Experimental Results and Analysis

We perform experiments to evaluate the feasibility and efficiency of our proposed SSA-SpiNNaker model, especially in terms of energy consumption, and accuracy. We deploy our proposed SSA-SpiNNaker model on the SpiNNaker hardware. After deployment, a large number of spiking neurons are generated, using various simulation modes, which are supported by the SpiNNaker hardware. Then, we compute the accuracy of our proposed SSA-SpiNNaker model with each simulation mode and compare the accuracy results with that of our pre-trained ANN sentiment analysis model. Detailed experimental results and analysis on the above performance metrics are presented in subsequent sections. In addition, from our investigation on existing works, we could not find any models in the published literature that provided an SSA-SpiNNaker model similar to ours. Hence, in this paper, we do not report any direct performance comparisons with the existing models.

### 4.1. Dataset Description and Usage

For our experiments, we use the Internet Movie Database (IMDB) dataset that contains movie reviews together with the binary sentiment polarity labels associated with every movie [53]. This IMDB dataset was created by a Stanford researcher, has the typical accuracy of 88.89%, and is widely used for several applications. The IMDB dataset used for the sentiment classification consists of 50,000 movie reviews from the users of IMDB. Every user labeled the review as either positive (represented by 1) or negative (represented by 0). Each review undergoes preprocessing and is encoded into a sequence of integer word indexes. The words in the reviews are usually indexed using the overall frequency of the word within the dataset. For instance, the integer ”3” encodes the third most frequent word in the data. Out of the 50,000 reviews, the data are divided into 25,000 reviews for training and 25,000 reviews for testing. The Keras model provides access to the IMDB dataset by default; thus, there is no need to download it separately for experiments. The method **keras.datasets.imdb.load data ()** is used to load the data from IMDB in the format that is required to train the neural network models.

### 4.2. Model Execution

After loading the IMDB movie review dataset, the ANN model is created, then trained and compiled. From the ANN model, the SNN model is created using various SpiNNaker simulators. In this case, using the INI simulator, we observe better results, especially in terms of accuracy, compared to other simulators. As a result, for all of our experiments we used the INI simulator as our default simulator. The SNN model used for our proposed SSA-SpiNNaker model is loaded with a total of 505,201 trainable parameters, and 0 non-trainable parameters. Then, the parsed SNN model is compiled. The details of our SNN model are presented in Table 5.

In our proposed SSA-SpiNNaker model, a total of 1,010,251 operations are performed for both the training and inference, by using 151 neurons and 505,050 synapses, with an average spike rate of 0.0091 spikes per simulation time step. In this case, five test samples are taken to evaluate the parsed model, where the top-1 and top-5 accuracies are observed as 100%. These top-1 and top-5 are standard accuracy matrices utilized to evaluate the performance of classification models. Typically, top-1 accuracy provides insight into the precision of the model, whereas top-5 accuracy provides insight into the model’s ability to handle uncertainty and diverse possibilities. Using the INI simulator, in general, our SNN model achieves 100% accuracy on five test samples, whereas our pre-trained ANN model only achieves 90% accuracy. In Table 6, we present the network summary of the SNN created for our proposed SSA-SpiNNaker model. 

For our proposed SNN model, the input layer represents the shape of the input, whereas the dense layers are fully connected layers with neurons connecting to every neuron in the previous layer.

### 4.3. Testing Our SSA-SpiNNaker Model

**Figure 4:**
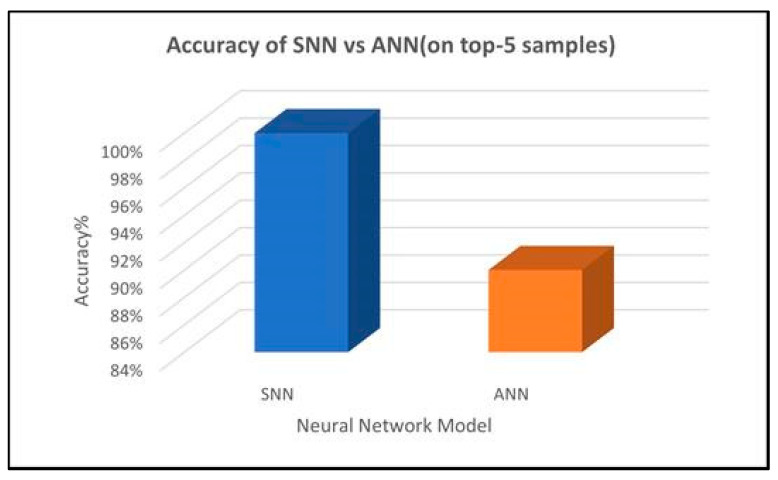
Accuracy of our proposed SNN vs. ANN for top-5 samples.

We tested our proposed SNN model utilizing different user review inputs. In this case, our SNN model achieves 100% accuracy. Figure 4 illustrates the accuracy of our pre-trained ANN model and our proposed SNN model for top-5 samples. These accuracy results are obtained using the INI simulator. As illustrated in Figure 4, for top-5 samples, our SNN model achieves 100% accuracy, whereas our pre-trained ANN model achieves 90% accuracy. The reason for utilizing top-5 samples for our proposed model is mainly because top-5 accuracy is considered as the correct prediction, if the true label is among the model’s top-5 predicted sentiments. In general, top-5 accuracy is utilized when managing diverse and complex datasets comprising multiple valid input classes. Although top-5 samples accuracy is typically considered to be sufficient to obtain the accuracy of the model. As future work, we will explore ways to obtain general accuracy by adopting statistical measures, such as top-N, where *N* is generic, in order to further enhance our proposed model.

**Figure 5:**
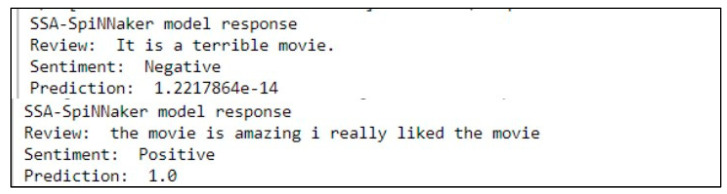
Response of our SSA-SpiNNaker model during testing phase on SpiNNaker hardware: for worst-case and best-case user inputs; in here, 1.2217864e-14 = 1.2217864 × 10^−14^.

In Figure 5, we present the response of our SSA-SpiNNaker model for various user inputs, especially for the worst-case and best-case inputs. As depicted, based on the user input words, for instance, for the worst-case (i.e., terrible…) and for the best-case (i.e., amazing…) inputs, our proposed model predicts the sentiment as a negative review and as a positive review, respectively.

**Figure 6:**
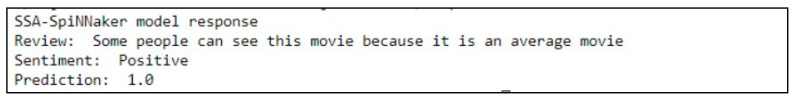
Response of our SSA-SpiNNaker model during testing phase on SpiNNaker hardware: for average user inputs.

We also tested our proposed SNN model for the average user inputs/review. As demonstrated in Figure 6, since the average review is not negative, our SSA-SpiNNaker model considers this average input as a positive review.

### 4.4. Evaluating Our SSA-SNN Model

**Figure 7:**
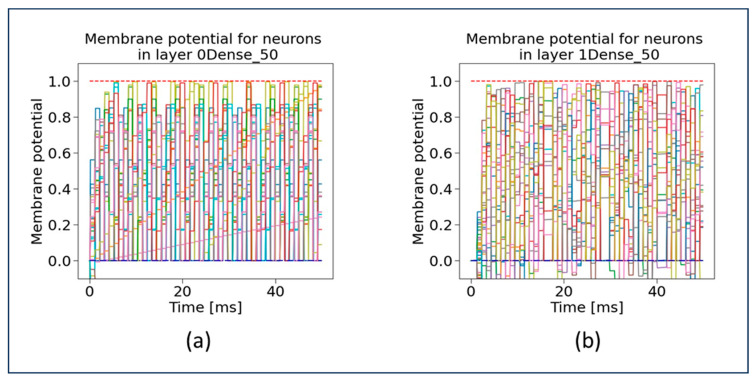
Membrane potential of neurons in SSA-SNN model at (**a**) 0Dense 50 layer and (**b**) 1Dense 50 layer.

In this subsection, we present the evaluation of our proposed SSA-SNN model using important characteristics. In our research work, we mainly focus on analyzing the neurons membrane potential in “0Dense 50 layer” of the SSA-SNN model. In this case, the membrane potential refers to the electrical potential difference across the cell membrane of neurons in “0Dense 50 layer”. The overall information processing of our SSA-SNN model neural network is determined by performing computations based on the activation function, with the inputs received from the previous layer neurons. Figure 7 demonstrates the membrane potential graphs for “0Dense 50 layer” and “1Dense 50 layer”, which explains the connectivity and activation patterns of the neurons. The blue scattered line (on the bottom) and red scattered line (on the top) in these graphs represent the reset (or resting) potential and firing threshold potential, respectively. Once a neuron reaches the firing threshold, it will automatically be set to the resting potential. At the resting potential, the neuron will reset, recover, and prepare for future firing. When the membrane potential of the neuron is at its threshold, it indicates that the neuron has received sufficient input and is ready for transmitting an electrical signal to its downstream connections. The firing threshold hold also determines when a neuron becomes active and initiates the output signal. The neurons voltage trajectory under different stamps is shown in the graphs (in Figure 7). The various points in the graph that lie between the reset potential and the firing threshold indicate that the neurons have not received enough input activation and are waiting for an opportunity to reach the firing threshold potential.

**Figure 8:**
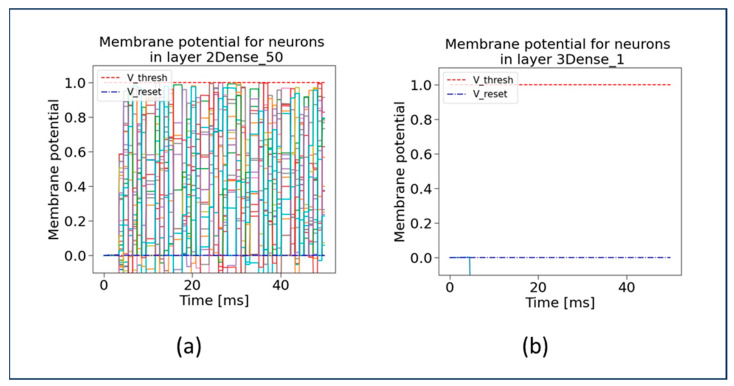
Membrane potential of neurons in SSA-SNN model at (**a**) 2Dense 50 layer and (**b**) 3Dense 1 layer.

Similar graphs (as in Figure 7) are generated and depicted in Figure 8 for the membrane potential for 2Dense 50 layer, and 3Dense 1 layer. As depicted in Figure 8b, the single neuron present in the 3Dense 1 layer is below the reset potential, which indicates that the neuron just finished firing a spike during the refractory period and is unable to fire a spike at that specific time. From the graph in Figure 8b, it is evident that only one neuron and the membrane potential refer to the single neuron.

**Figure 9:**
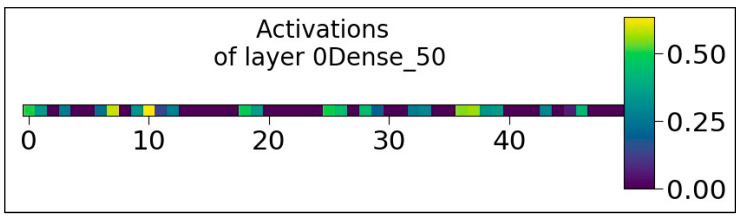
Activation values of the 50 neurons belonging to 0Dense 50 layer.

**Figure 10:**
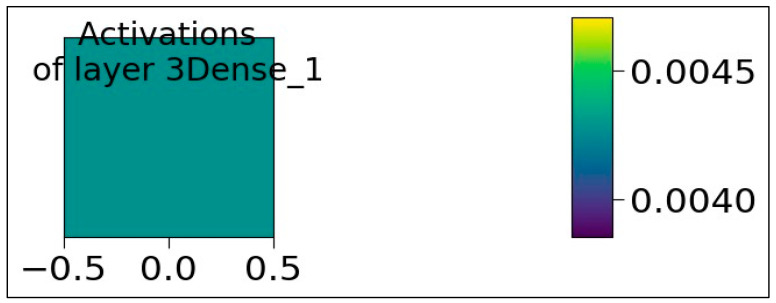
Activation values of the single neuron belonging to 3Dense 1 layer.

Figure 9 shows the Activation map values of 50 neurons at “0Dense 50 layer” while processing input data. Visualization of the activation map will provide insight into the internal representations for specific input for each of the layers in the model. The values 0.00, 0.25, and 0.50 in Figure 9 represent the activation values. As shown in each layer, the activation values are represented with various colors. The activation values at layer 0 (in green) show the moderate activation values, and signify a moderately strong activation within the activation map. The activation values at layer 10 (in yellow) represent the high activation values and indicate that the neurons at layer 10 have strong activation. Similarly, the activations at layer 20 (in blue) indicate the lower activation values. The high, moderate, and low activations at various layers indicate how the neurons are activated at a particular layer, having a significant influence on the network output. The graph in Figure 10 shows the activation values of the single neuron at “3Dense 1 layer”. In this case, there is only one neuron in the layer with moderate activation values.

**Figure 11:**
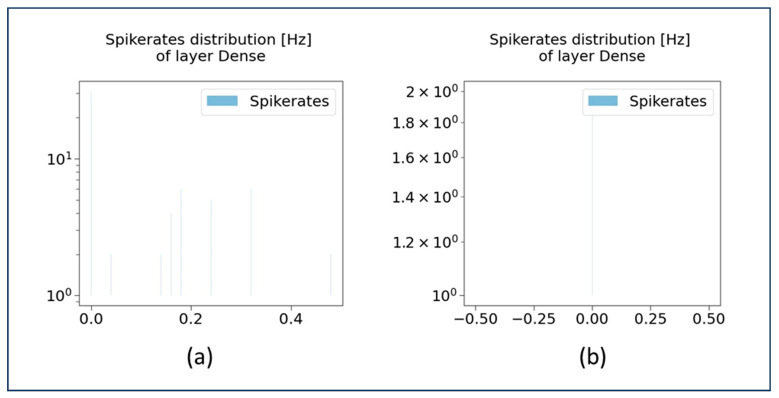
Spike rate distribution of neurons at (**a**) 0Dense 50 layer and (**b**) 3Dense 1 layer.

The spike rate distributions at 0Dense 50 layer and 3Dense 1 layer are illustrated in Figure 11. These graphs (in Figure 11) demonstrate how the spike rates are distributed within the dense layer, under different frequencies (in Hz) (in y-axis) as well as at different activation values (in x-axis). The frequency of spike rates is usually represented in the powers of 10; for instance, the frequency 1.2 × 10^0^ is usually read as 1.2 Hz. As depicted in Figure 11b, the single neuron in the 3Dense 1 layer is activated with the activation value 0.0, indicating that the neurons activation value is at its lowest at a spike rate frequency of 2 Hz.

**Figure 12:**
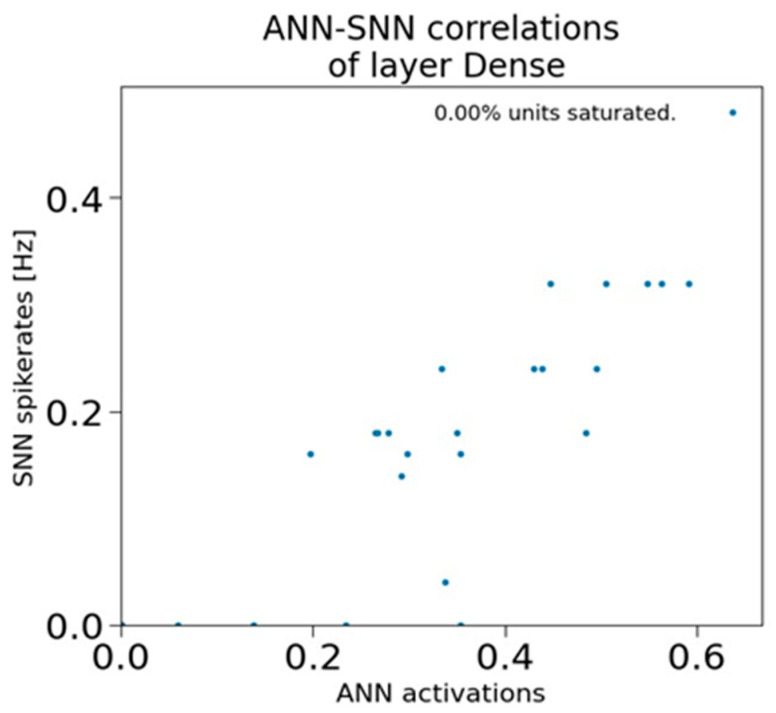
Correlation between ANN activations vs. SNN spike rates.

**Figure 13:**
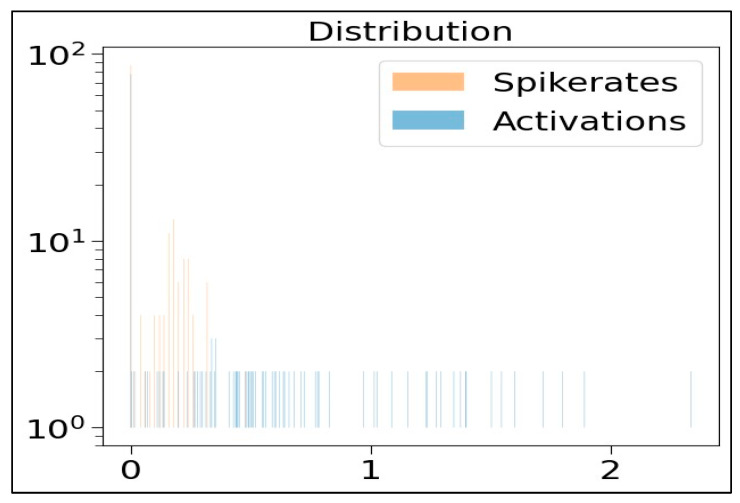
Activity distribution of ANN activations and SNN spike rates.

In Figure 12, the graph demonstrates the comparison of ANN activations versus the SNN spike rates at 0Dense 50 layer. This graph (in Figure 12) shows the representations associated with the dense layers of ANN and SNN networks. It also illustrates the correlation between the SNN spike rates and ANN activations averaged over one sample. In Figure 12, the data points in graph represent the correlation values between the ANN and SNN outputs, used to indicate the similarity between the ANN and SNN. In this case, the clustered blue dots suggest a strong correlation between ANN and SNN outputs, whereas the scattered data points represent a weaker correlation. The graph in Figure 13 depicts the activity distribution of ANN activations and SNN spike rates distributions across various dense layers. In Figure 13, the x-axis represents the 0, 1, and 2 Dense layers, and the y-axis represents the frequency. From this graph (in Figure 13), it is evident that at the 0Dense 50 layer, the spike rates (represented in orange lines) are more compared to the ANN activations (represented in blue lines). This is mainly because at the 0Dense layer, the input is converted into spikes, in order to be processed by the neurons in SNNs.

**Figure 14:**
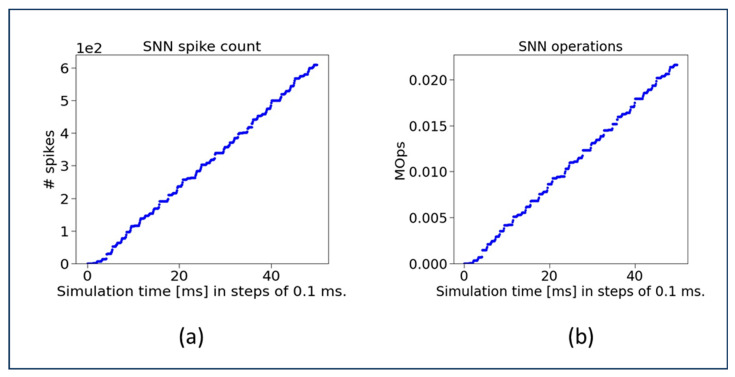
(**a**) SNN spike count for a time step of 0.1 ms; in here, # spikes means number of spikes, and 1e2 = 1 × 10^2^; (**b**) Total number of SNN operations for a time step of 0.1 ms.

In Figure 14a,b, the graphs demonstrate the total number of SNN operations and the spike counts, respectively, which take place over a 0.1 ms time step. The graph (in Figure 14a) depicts the number of spikes generated at various time stamps, whereas the graph (in Figure 14b) shows the number of SNN operations created under various times stamps during the execution of our proposed SSA-SNN SpiNNaker model.

### 4.5. Energy Consumption

Energy consumption is also an important performance metric utilized to evaluate our proposed SNN model. Due to the computational resource limitation, the energy consumption for our pre-trained ANN model is not calculated. However, from our investigation on the existing works on ANNs and SNNs, it was revealed that SNNs consume less energy than ANNs. 

**Figure 15:**
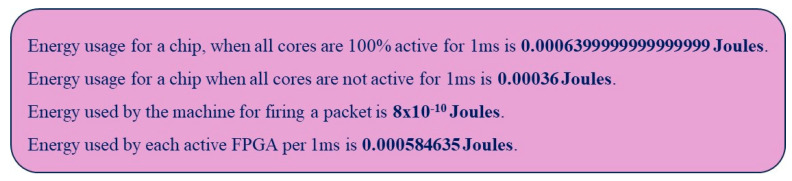
Energy consumption report during the execution of our SSA-SpiNNaker model.

**Figure 16:**
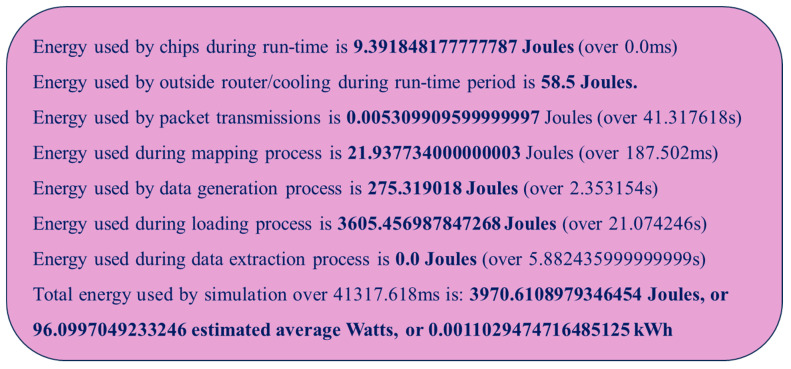
Energy consumption summary report during the execution of our SSA-SpiNNaker model.

The detailed energy consumption report as well as the summary report, during the execution of our proposed SSA-SpiNNaker model, are presented in Figure 15 and Figure 16, respectively. As demonstrated in both figures, our proposed SSA-SpiNNaker model consumes energy in various ways while being executed. These two figures illustrate how energy is utilized by different parts of the SpiNNaker platform for different tasks when executing our proposed SSA-SpiNNaker model. For instance, the chip as well as the routers consume energy during runtime. The loading process has the highest energy consumption. Furthermore, as in Figure 16, the mapping, data packet transmission, and data generation processes also consume energy, whereas the data extraction process does not consume any energy. In total, our proposed SSA-SpiNNaker model consumes 3970 Joules of energy, while processing around 10,000 words and predicting either a positive or a negative review.

## 5. Conclusions and Future Work

In this paper, we introduced a novel, unique, and efficient neuromorphic NLP sentiment analysis model based on the SNNs, deployed on the SpiNNaker neuromorphic platform. Our goal was to enhance the NLP traits/abilities, by harnessing and integrating the SNNs traits, as well as deploying the integrated solution on neuromorphic hardware. Our proposed SNN-based sentiment analysis model was created in such a way: to be energy efficient and also to be faster than the ANN-based NLP models, while predicting the sentiment. Our proposed SNN model, converted from our ANN model, is trained and deployed on the SpiNNaker hardware, which enables leveraging energy efficiency, and inherent parallelism of SNNs.

Our proposed SSA-SpiNNaker model achieved 100% accuracy, and only consumed 3970 Joules of energy, while processing around 10,000 words and predicting a positive/negative review. From these results and analysis, it is evident that parallel processing capabilities and low energy consumption, associated with our proposed SNN model, are indeed a promising avenue for NLP tasks, compared to the traditional ANN-based NLP models. Our research also clarifies the importance of our proposed SNN model by considering the neural dynamics and demonstrates that the spike-based computations impact the effectiveness of the overall NLP tasks. Furthermore, from our investigation on existing works, we could not find any models in the published literature that provided an SSA-SpiNNaker model similar to ours, demonstrating the uniqueness of our proposed model.

As future work, we will further enhance our proposed SNN model and explore the applicability of SNNs to various NLP tasks, such as neural embeddings, as well as to tasks that are beyond sentiment analysis. Also, as future work, we are planning to introduce field programmable gate arrays (FPGAs)-based hardware architectures for our proposed models. This is mainly because our previous work and analysis ([54,55,56,57,58]) demonstrate that FPGAs are one of the best avenues to deploy/execute compute and data-intensive applications, such as SNNs, on resources-constrained devices. We will also incorporate dynamic reconfiguration techniques [59,60] to create dynamic reconfigurable architectures (similar to [61,62,63]) to integrate adaptability traits to our proposed models.

## Figures and Tables

**Table 1:** SNNs and ANNs comparison.

Parameters	Spiking Neural Network (SNN)	Artificial Neural Network (ANN)
Neuron [9]	Spiking Neuron	Artificial Neuron
Representation of Information [9]	Spike trains	Scalars
Mode of Computation [9]	Differential Equations	Activate function
Topology [9]	RSNN, SCNN, Hopfield network, and LSM	RNN, CNN, DBN, LSTM and DNC
Features [9]	Real time, low power, online learning, parallel data processing	Online learning, moderate parallelization

**Table 2:** Tabular comparison of various SNN models.

Model	Number of Floating-Point Operations (FLOPS)	Number of Variables	Computational Complexity
Integrate-and-Fire Model	5	1	Very Low
Hodgkin–Huxley Model	1200	1	High
Izhikevich Model	13	2	Very Low
Spike Response Model	50	1	Low

**Table 3:** Characteristics of existing neuromorphic hardware platforms.

Platform	Technology (mm)	Electronics	Chip Area (mm^2^)	Neuron Model	On-Chip Learning	Neuron Number (Chip)	Synapse Model	Synapse Number (Chip)	Online Learning	Power
TrueNorth [9]	ASIC-CMOS 28	Digital	430	LIF	No	1 Million	Binary 4 modulators	256 M	No	65 mW (per chip)
BrainScaleS [9]	ASIC-CMOS 180	Analog/Digital	50	Adaptive exponential IF	No	512	Spiking 4-bit digital	100 K	Yes	2 kW Per module (peak)
NeuroGrid [9]	ASIC-CMOS 180	Analog/Digital	168	Adaptive Quadratic IF	No	65,000	Shared dendrite	100 M	Yes	2.7 W
Loihi [9]	ASIC-CMOS 14 nm	Digital	60	LIF	Yes (with plasticity rule)	131,000	N/A	126 M	Yes	0.45 W
SpiNNaker [9]	ASIC-CMOS 130 nm	Digital	102	LIF LZH HH	Yes (synaptic plasticity rule)	16,000	Programmable	16 M	Yes	1 W (per chip)

**Table 4:** Characteristics of various memory access of SpiNNaker chip.

Memory Area	Size	Visibility	Speed/CPU
SDRAM [11]	128 MB	Node-Local	64 MBps
SRAM [11]	32 KB	Node-Local	25 MBps
ITCM [11]	32 KB	Node-Local	800 MBps
DTCM [11]	64 KB	Node-Local	800 MBps

**Table 5:** Details of the SNN.

Number of Operations	Number of Neurons	Number of Synapses
1,010,251	151	505,050

**Table 6:** Network summary table of the SSA-SpiNNaker SNN model.

Layer (Type)	Output Shape	Param #
input (Input Layer)	(5, 10,000)	0
0Dense 50 (Dense)	(5, 50)	500,050
1Dense 50 (Dense)	(5, 50)	2550
2Dense 50 (Dense)	(5, 50)	2550
3Dense 1 (Dense)	(5, 1)	51

## Data Availability

Not applicable.
